# Prevalence, Genetic Diversity, and Quantification of the RNA Genome of the Hepatitis E Virus in Slaughtered Pigs in Serbia

**DOI:** 10.3390/ani14040586

**Published:** 2024-02-10

**Authors:** Lazar Milojević, Branko Velebit, Vesna Janković, Radmila Mitrović, Nikola Betić, Sara Simunović, Mirjana Dimitrijević

**Affiliations:** 1 Institute of Meat Hygiene and Technology, Kaćanskog 13, 11040 Belgrade, Serbia; branko.velebit@inmes.rs (B.V.); vesna.jankovic@inmes.rs (V.J.); radmila.mitrovic@inmes.rs (R.M.); sara.rajic@inmes.rs (S.S.); 2 Faculty of Veterinary Medicine, University of Belgrade, Bulevar Oslobođenja 18, 11000 Belgrade, Serbia

**Keywords:** foodborne viruses, hepatitis E virus, zoonosis, slaughterhouse, Serbia

## Abstract

**Simple Summary:**

In surveillance studies conducted in industrialized EU nations, a rise has been noted in hepatitis E infections that are unrelated to travel. According to previous research studies, eating undercooked or uncooked meat and organs, as well as products from pigs or other wild animals (wild boars and deer), was predominantly linked to human hepatitis caused by the hepatitis E virus genotype 3. Furthermore, domestic pigs and wild boars represent nature’s primary reservoirs of this virus, and the liver is the main organ for hosting this virus.

**Abstract:**

The goal of this study conducted in Serbia was to detect HEV in pig liver samples from slaughterhouses, retail outlets, and environmental swabs over the course of a year. All positive HEV samples were measured and expressed as HEV gene copy numbers per gram of sample, and a representative number of samples were sequenced using the Sanger approach. A total of 45 HEV-positive samples were re-amplified using nested RT-PCR employing CODEHOP primers targeting ORF2 (493 nucleotides). The average prevalence of the HEV genotype 3 in all pig liver samples from the slaughterhouses was 29%, while HEV prevalence was 44% in liver samples from animals younger than 3 months. HEV RNA was found in thirteen out of sixty (22%) environmental swab samples that were taken from different surfaces along the slaughter line. Our findings confirmed seasonal patterns in HEV prevalence, with two picks (summer and winter periods) during the one-year examination. Among HEV-positive samples, the average viral particles for all positive liver samples was 4.41 ± 1.69 log_10_ genome copies per gram. Phylogenetic analysis revealed the majority of HEV strains (43/45) from Serbia were grouped in the HEV-3a subtype, while two strains were classified into the HEV-3c subtype, and one strain could not be classified into any of the HEV-3 subtypes.

## 1. Introduction

Hepatitis E virus (HEV) is a member of the Hepeviridae family, genus Orthohepevirus [1], and it is the leading cause of viral hepatitis in humans worldwide [2]. Furthermore, four Orthohepevirus species have been identified, classified as Orthohepevirus A to Orthohepevirus D. The most well-studied type is Orthohepevirus A, which has seven genotypes, but only the first four (1–4) can infect people [3]. Genotypes 1 and 2 typically infect humans through water, and this path of infection is common in underdeveloped nations [4]. In addition, genotypes 3 and 4 are widespread in humans and animals and are especially common in developed countries [5]. HEV is now classified as an emerging zoonotic disease [2].

Food viruses cause foodborne infections and are a developing and substantial global problem, having a considerable impact on economic growth in countries throughout the world [6]. The European Food Safety Authority (EFSA) paid special attention to foodborne viruses in a 2015 report [7]. According to the EFSA, viruses are the most common cause of foodborne disease outbreaks. The human incidence of infection caused by HEV, particularly genotype 3 strains, has been increasing in several countries. According to the European Centre for Disease Prevention [8], the number of HEV infections has increased from 514 in 2005 to 5617 in 2015. Additionally, strong sequence identity has been found between strains circulating in pig herds and those isolated from people [9]. Several studies have found a significant incidence of anti-HEV antibodies in pigs on pig farms, indicating the importance of this animal species as a natural reservoir of this virus [10]. Pigs infected with HEV normally do not show clinical signs, although in rare situations, a mild to moderate, acute, self-limiting liver infection might occur [11]. The most typical route of HEV transmission in pigs is fecal–oral, and the liver is the target organ, although the virus has been identified in other organs. Unfortunately, because diagnosing HEV infection with ante-mortem and post-mortem investigations is not possible, infected pigs entering abattoirs are judged as healthy, and their meat and other tissues are used for further production. Speaking of pork, this is the most common meat type in the world, with 106,103,000 tons produced in 2021 according to United States Department of Agriculture Foreign Agricultural Service [12], and so requires a thorough examination of the potential risks of HEV infection to humans. As previously stated, liver tissue is the target organ, and so meat products containing liver are the most obvious sources of foodborne HEV.

The majority of HEV-infected people have no visible symptoms, and the sickness progresses asymptomatically [13]. However, some individuals develop abdominal pain, vomiting, fever, icterus with nausea, and hepatomegaly after an incubation period of 14 to 56 days [14]. Around 2% of HEV-caused human cases result in death [14].

The purpose of this study was to detect and characterize HEV strains in pig liver samples from slaughter lines, unrelated retail outlets, and in slaughterhouse environmental swabs, throughout a one-year period in Serbia. In addition, the prospective seasonal patterns of HEV incidence in pigs were investigated, as well as the spatial distribution of HEV in three different epidemiological regions in Serbia. HEV-positive samples were quantified and expressed as HEV copy numbers, and a representative number of viruses were sequenced using the Sanger approach.

## 2. Materials and Methods

### 2.1. Study Design

In Serbia, sampling was conducted over a one-year period (January to December 2020). The sampling was conducted quarterly throughout the year, covering all four seasons (winter season: January–March; spring season: April–June; summer season: July–September, and autumn season: October–December).

In total, 1020 samples collected from three regions in Serbia (Table 1) comprised pig liver samples from slaughter lines (N = 900), environmental swabs from the same slaughter lines (N = 60), and unrelated pig livers from retail outlets (N = 60). All samples were collected from one slaughterhouse in each district. Liver samples were collected from 75 clinically healthy pigs at random in each season in all three regions (Table 1). The samples were divided into two age categories: pig less than three months old (50 samples); and pig between 6 and 12 months old (25 samples).

At slaughterhouses, pig livers were removed from the carcasses and sterile scissors, forceps, and disposable materials were used to excise samples from the liver lobe beneath the gallbladder following its removal. The sample weight was approximately 25 g. All samples were deposited in sterile 50 mL Falcon conical centrifuge tubes, transported to the laboratory within 6 h, and maintained at −20 °C in the freezer until analysis.

Five environmental swabs were collected during each slaughterhouse visit from various sites/surfaces on the slaughter line that had contact with offal during manipulation (inspection tables, sharpeners, knives, offal collection containers, and hooks for hanging livers). Each surface was sampled with an environmental swab (Copan, Brescia, Italia) dampened in 5 mL of Dulbecco’s Modified Eagle’s Medium (Sigma-Aldrich, Steinheim, Germany).

Furthermore, every season, 15 pig livers were randomly gathered from retail establishments located within the same geographical regions, independently of all other liver samples and without any association with the samples obtained at slaughter.

Until RNA extraction, the samples were stored at −20 °C.

### 2.2. Sample Preparation and Nucleic Acid Extraction

Liver samples weighing approximately 100 mg were inoculated with 10 µL of process control virus (Mengovirus, vMC0, 2.1 × 10^5^ TCID50). Next, each sample was homogenized in 1 mL of Trizol reagent (Invitrogen, Carlsbad, CA, USA) and 600 µg of zirconia beads using the BeadBeater homogenizer (Biospec, Bartlesville, OK, USA) for 3 min. Subsequently, 200 µL of chloroform was added, and the mixture was vortexed for 2 min. The samples were incubated for 10 min at room temperature before being centrifuged at 12,000× *g* for 10 min at 4 °C. Three different phases were separated using phase lock heavy gel tubes (5 Prime, Hamburg, Germany). The upper aqueous phase was retained and stored at −20 °C until further RNA extraction. Total viral RNA from samples was investigated using a commercial RNeasy Mini Kit (Qiagen, Hilden, Germany) according to the manufacturer’s instructions, aliquoted, stored at −20 °C, and thawed only once.

The swabs from the surfaces were vortexed for 60 s and centrifuged for 15 min at 2500× *g*. Nucleic acids were extracted from the supernatants using the above-mentioned RNeasy Mini Kit (Qiagen, Hilden, Germany).

### 2.3. Detection of HEV RNA

A quantitative reverse transcription PCR (RT-qPCR) assay was used to detect the HEV3 genotype using previously described primers and probes [15], but the primer HEV3-f was modified. At position 5323, the degenerate nucleotide “R” was substituted for the guanine (G) (nucleotide position determined based on the HEV genome registered in GenBank under accession number AF060669). RT-qPCR was performed in a 25 μL reaction mixture on an AriaMx Real Time PCR machine (Agilent, Santa Clara, CA, USA) while the following thermal profile was used: 1 cycle at 55 °C for 60 min and 95 °C for 5 min, followed by 50 cycles of 95 °C for 15 s, 60 °C for 60 s, and 65 °C for 60 s. For every reaction, 5 μL of extracted RNA were used. Both positive and negative controls were used in each qPCR run. Results with a cycle threshold value (Ct) lower than 40 were considered to be HEV positive, and results with higher Ct values were interpreted to be HEV negative. The efficiency of extraction was calculated using an RNA standard curve for vMCO [16]. The minimum acceptable sample recovery was set to ≥3%.

### 2.4. Quantification of HEV Positive RNA Samples

A synthetic molecule (RNA transcript) was constructed in order to determine the number of genome copies per gram of the virus in the positive liver samples. DNA complementary to HEV (GenBank acc. No. MG051653), size 71 nucleotides, was cloned into both the pEXA2 vector (Eurofins, Hamburg, Germany) and *E. coli* One Shot aTOP10F (Invitrogen, Carlsbad, CA, USA). The plasmid obtained was linearized using HindIII-HF enzyme (NEB, Ipswich, MA, USA) and purified using an MinElute purification kit (Qiagen, Hilden, Germany). DNA was transcribed using a Riboprobe System T7 transcription kit (Promega, Madison, WI, USA). The target sequence was used to generate the standard curve, but only curves with a slope between −3.1 and −3.6 and a R2 ≥ 0.98 were used further for quantification.

### 2.5. Phylogenetic Analysis

We selected isolates of the liver samples from all regions and all seasons. A total of 45 HEV-positive samples were re-amplified using a nested RT-PCR with CODEHOP primers targeting ORF2 to produce a final 493-nucleotide fragment for Sanger sequence analyses [17,18]. These fragments were typed according to Smith et al. [19]. Using 1.5% agarose gel dyed with SYBR Safe Dye (Invitrogen, Carlsbad, CA, USA), the nested RT-PCR results were visualized. Following the manufacturer’s instructions, bands of the expected size were removed, and cDNA was purified using Wizard SV Gel and the PCR Clean-Up System (Promega, Madison, WI, USA).

The evolutionary history was inferred by using the maximum likelihood method employing Hasegawa–Kishino–Yano+G as the nucleotide substitution model. Bootstrap values greater than 70% acquired after 1000 replications were used. Initial tree(s) for the heuristic search were obtained by applying the Neighbor-Joining (NJ) method to a matrix of pairwise distances estimated using the maximum composite likelihood (MCL) approach. The trees were drawn to scale, with branch lengths measured according to the number of substitutions per site. Evolutionary analyses were conducted in MEGA11 [20]. The obtained sequences have been submitted to GenBank, with the accession numbers listed in Table S1.

### 2.6. Statistical Analysis

Fisher’s exact test and the chi-square test were employed to examine the significance of differences in the prevalence values. Using the Shapiro–Wilk test, the normal distribution of the number of genome copies per gram (g.c./g) was evaluated. Due to the non-normal distribution of the samples (Shapiro–Wilk test, *p* 0.05), a Kruskal–Wallis analysis of variance was used to compare the groups. In cases where there was a statistically significant difference between the groups, Dunn’s test was used to compare the groups.

Statistical analyses were performed using GraphPad Prism version 9 for Windows (GraphPad Software, San Diego, CA, USA), www.graphpad.com, accessed on 11 September 2023, and Microsoft Excel version 2110.

## 3. Results

### 3.1. Molecular Detection and Prevalence of HEV RNA

In this study, among the 900 tested liver samples collected at slaughterhouses, a total of 261 HEV-positive samples were discovered (prevalence, P = 29%). In terms of animal-age categories, HEV was not detected in the livers of pigs aged 6 to 12 months, so all detected 261 HEV-positive samples came from piglets younger than 3 months (P = 44%). Furthermore, there was no statistically significant difference in the HEV detection rates depending on sex (448 male pigs studied yielded 130 positive results, whereas 452 female pigs yielded 131 positive results; *p* > 0.999). In contrast, there were statistically significant differences across the three examined geographical regions (*p* < 0.001). The detection rate of HEV in all the liver samples from slaughter lines was highest in the Srem region (49%) and lowest in the Šumadija region (4.67%). The HEV detection rate in the livers from the Kolubara region was 33.33%. During the study, there were two peaks (winter and summer) where the detection rate of HEV was higher (33.89% and 37.33%) than in the other two seasons (22.69% and 23.11%), respectively.

Statistically significant differences (*p* < 0.05) were found among the winter/spring and winter/autumn periods, while the statistical significance among summer/autumn was at the level of *p* < 0.01 and among summer/spring was at the level of *p* < 0.001. Since positive samples were only detected in pigs younger than 3 months, we statistically analyzed this group separately. The results showed even more significant statistical differences, so among summer/spring and summer/autumn, they were at the level of *p* < 0.001, among winter/spring the significance was at the level of *p* < 0.01, and among winter/autumn, the difference was at the statistical level of *p* < 0.05.

Thirteen of the sixty (22%) environmental swab samples collected from various surfaces along the slaughter line harbored HEV RNA. In the Srem region, ten swabs (50%) held viral RNA, whereas in the Kolubara region, just three swabs were positive (15%). In contrast, HEV RNA was not detected from the swabs of slaughter line equipment in the Šumadija region throughout this study. We examined the seasonality of the environmental samples between seasons, but no statistically significant variations were found.

Viral RNA was detected in three (5%) of the liver samples taken at retail from the three regions. Two of the three positive liver samples were collected in winter, while the third was obtained in spring. HEV was not detected in livers at retail that were collected throughout the summer and autumn seasons.

### 3.2. Quantification of Positive HEV RNA Samples

The average viral particles for all positive liver samples was 4.41 ± 1.69 log_10_ g.c./g. With regard to the seasons, the median HEV loads were similar and without statistically significant differences. On the other hand, there were statistically significant differences in the viral particles in livers between regions. The median HEV load in the livers from Srem was 4.30 log_10_ g.c./g, i.e., ranging in the first and third quartiles from 3.57 to 5.76 log_10_ g.c./g. Kolubara and Šumadija regions had median HEV loads of 3.74 and 2.94 log_10_ g.c./g, respectively. Therefore, viral particles were significantly higher in pig livers collected from Srem than in those collected from Kolubara and Šumadija (*p* < 0.01).

### 3.3. Phylogenetic Analyses

The phylogenetic analysis compared partial ORF2 RNA sequences (493 bp) from forty-five Serbian HEV strains with 47 HEV reference sequences. The reference sequences represented classic genotypes 1, 2, 3, and 4, as well as novel genotypes 5, 6, and 7, as described by Smith et al. [19]. The reference sequences were obtained from several host species and originated from diverse countries in Asia, Africa, America, and Europe.

**Figure 1:**
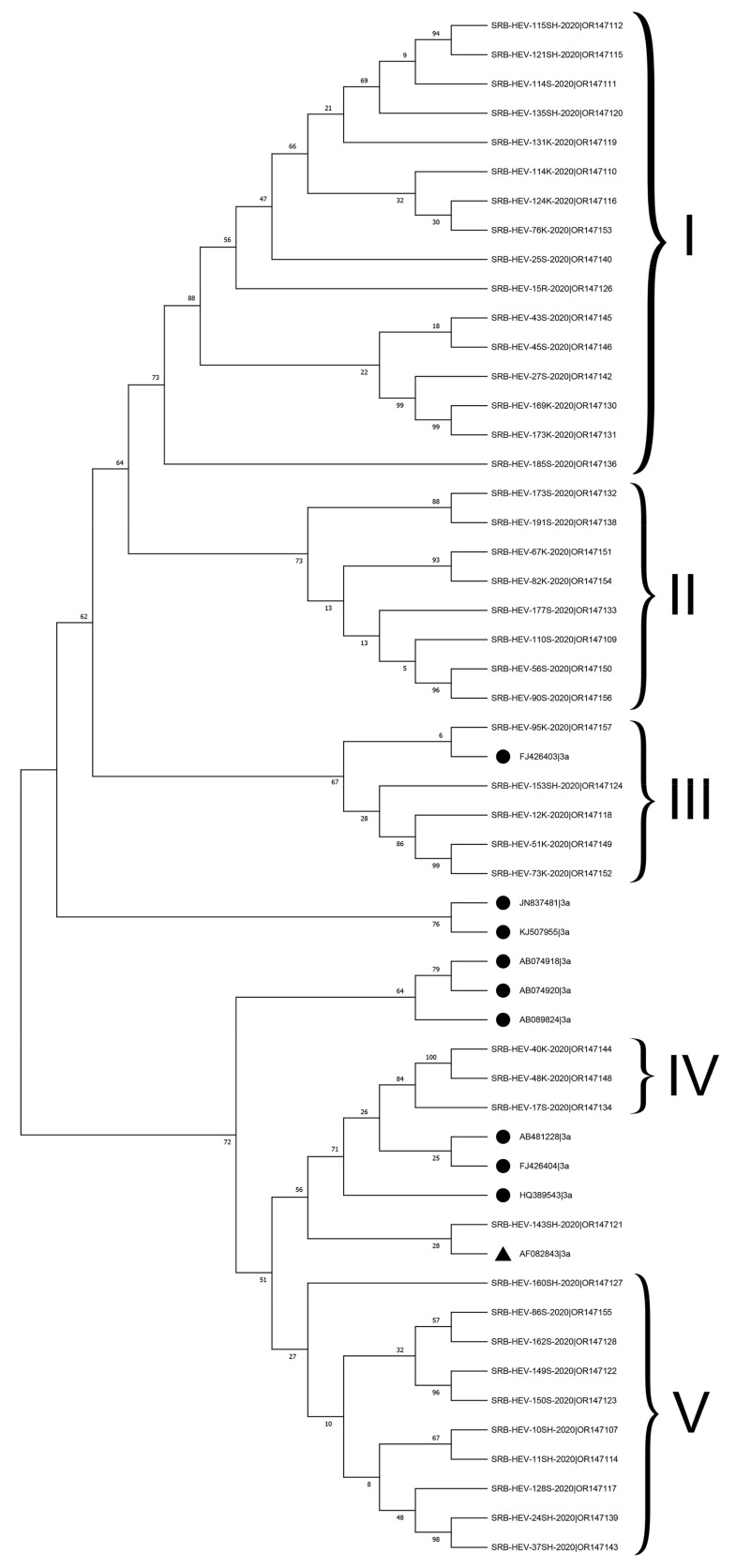
Phylogenetic comparison of HEV-3a subtype RNA sequences from this study against reference sequence AF082843 (▲) and HEV-3a sequences from other countries (●). The phylogenetic tree was constructed using capsid 493 bp-length partial sequences within the HEV ORF2 fragment using a maximum likelihood analysis employing Hasegawa–Kishino–Yano+G as the nucleotide substitution model. Bar length represents a rate of 0.1 substitutions/site. Bootstrap values greater than 70% acquired after 1000 replications are shown. HEV-3a RNA sequences obtained in this study were deposited at the Genbank under the accession numbers indicated in Table S1.

The phylogenetic analysis revealed that all Serbian HEV sequences, which had 100% bootstrap support, were clustered within genotype HEV-3, with separate branches according to the different regions. Within this genotype, forty-three HEV RNA sequences were clustered unambiguously into the HEV-3a subtype, with nucleotide p-distances ranging from 0.055 to 0.112 for the HEV-3a reference strain (AF082843). Genetic and phylogenetic analysis of the Serbian HEV-3a subtypes showed that in the ORF2 HEV viruses formed five clusters (Figure 1).

Seven sequences (SRB-HEV-114K-2020, SRB- HEV-51K-2020, SRB-HEV-73K-2020, SRB-HEV-76K-2020, SRB-HEV-173K-2020, SRB-HEV-169K-2020, and SRB-HEV-110S-2020) showed higher p-distances (>0.103) to the HEV-3a reference strain (AF082843).

Additional examination of the HEV-3a subtype sequences, specifically comparing them to a group of reference HEV-3a strains from the USA (JN837481), Canada (KJ507955), Japan (AB074918, AB089824), Korea (FJ426403), and the UK (HQ389543), showed that 16 sequences from cluster I and 8 sequences from cluster II were not closely associated with any of these reference strains (with p-distances greater than 0.11). Five Serbian HEV-3a RNA sequences exhibited a close relationship with the Korean FJ426403 reference sequence, thereby constituting a distinct cluster III. Clusters IV, consisting of three sequences, and V, consisting of ten sequences, had limited similarities with clusters I–III.

In addition to HEV-3a, this research classified two Serbian HEV sequences (SRB-HEV-47R-2020|OR147147 and SRB-HEV-50K-2020) as subtype HEV-3c. However, the latter sequence was not deposited at the GenBank database because its ORF2 fragment size was too short. One RNA sequence (SRB-HEV-25SH-2020|OR147141) could not be assigned into any of the established subtypes of HEV-3 due to its significant divergence. However, sub-clustering phylogenetic analysis indicated possible assignment to the HEV3-h2 subtype.

## 4. Discussion

According to the official data [21], pork production makes up the largest production of total meat production in Serbia, and Serbs have a tradition of eating pork meat. There are currently few published data about HEV in Serbia, its presence in well-known natural reservoirs (mainly in domestic pigs), and the potential for human infection through the consumption of infected food. Because domestic pig livers are organs in which HEV are frequently found, and because this organ can be consumed by humans either directly or indirectly (as an ingredient in meat products), we looked for the presence of this virus in pig liver samples.

The detection rate of HEV in pig livers from slaughterhouses was 29%, according to this study’s findings. Similar findings were published in Europe by [22,23,24]. Similar results were found in Hungary, a country adjacent to Serbia, where 31% of the total pig liver samples tested positive for HEV [25].

In our study, liver samples were divided into HEV detection rate groups based on the age of the pigs. HEV was present in 44% of the livers of pigs younger than 3 months. Previous research by Milojević et al. [26] has demonstrated that the HEV prevalence in this pig age category was 34%. These are important data because a significant number of pigs under the age of 3 months are slaughtered due to the local pork consumption habits and culture in Serbia. Forgách et al. [25] observed a HEV prevalence of 36% for this age category of pigs in neighboring Hungary. Likewise, Fernandez-Barredo et al. [27] and Leblanc et al. [28] showed a high prevalence of HEV in Spain and Canada (41% and 52.9%, respectively). Ruggeri et al. [29] reported a prevalence of 32% in Italy, which was lower than our findings, but still considerably high. Similarly, Jackova et al. [30] have found a high HEV prevalence in this pig age group in Slovakia (29.6%), but their findings of HEV prevalence originated from testing rectal swab samples rather than livers. According to Raspor Lainscek et al. [31], the prevalence of HEV for this pig age group was substantially lower (12.1%) in Slovenia.

On the other hand, in our study, no HEV was detected in the liver of pigs older than six months throughout the course of a year. The same finding was obtained in a previous study carried by Milojević et al. [26]. The HEV prevalence in Slovenian pigs older than 6 months was rather low (0.25%), according to study carried by Raspor Lainscek et al. [31]. Nonetheless, published studies from several European countries demonstrate that there are differences in the prevalence of HEV in this older age group of pigs. In France, for example, Feurer et al. [32] found that HEV prevalence in pigs aged 6 months and older was 2.8%. Similar HEV prevalence rates in pigs of this age group were reported in other European countries: 4% in Germany [33], 5% in the Czech Republic [34], 6.5 percent in the Netherlands [35], and 3% in the UK [23].

Substantial differences in HEV detection rates between the two pig age groups examined corroborated the idea that after two months of passive immunity, piglets are the most susceptible to HEV infection. In addition, breeding sows have been identified as possible HEV reservoirs, capable of spreading the virus to piglets after the initial post-natal protective period [36]. The high detection rate of HEV in the livers of piglets up to three months of age in Serbia compared to other European countries could be explained by the low level of biosecurity on Serbian farms, and the fact that a large proportion of these animals likely came from small farms that house different types of pigs without proper separation. In addition, the breeding systems are extensive and facilitate cross- contamination between age groups. Hence, the conditions could be ideal for HEV infection of piglets and rapid, straightforward viral transmission between young animals during the early stages of production.

Alternatively, HEV-immune pigs entering slaughterhouses at 6 months or older no longer actively shed the virus, and it is highly unlikely that the virus would be discovered in their tissues. The research conducted by Lupulović [37] confirms this hypothesis. The HEV seroprevalence in fatteners from three farms in our country was 73.33%, according to her results. In developed EU countries, intensive breeding systems with much bigger numbers of pigs and high degrees of biosecurity controls are mostly used. After the end of passive immunity in intensive systems, considerably fewer piglets are infected. In contrast, such farms are more likely to have a greater number of infected pigs during the last phases of fattening, which could be one of the reasons why the frequency of HEV in this pig age category is higher in EU countries. Srem was the region with the highest HEV detection rate (49%), whereas Šumadija had the lowest (4.67%) proportion of HEV-positive pigs. This shows that the detection rate of HEV is not uniformly distributed across the three analyzed regions, but significantly differs. Similar patterns of HEV region-dependent prevalence in a single country were described in study by De Sabato et al. [38].

To study the probability of seasonal HEV infection, we collected and tested livers over the course of a year. Our data suggest that the virus is statistically more prevalent in the summer and winter seasons than in spring and autumn. Data on seasonal patterns in HEV are scarce, while published human studies have shown seasonal patterns of HEV infection primarily linked to environmental circumstances or weather disasters. During monsoon floods, a higher number of cases were reported in India, Nepal, and Pakistan [39]. According to Zhu et al. [40], compared with other annual seasons, the winter season in China had the highest number of acute human hepatitis cases caused by HEV. However, no seasonal impact on the frequency of disease was found in a 5-year study of human HEV infection cases conducted in France [41]. However, relatively few studies have looked into the possibility of a seasonal incidence of HEV in pigs. Lu et al. [42] looked at seasonal trends of HEV in domestic pigs. Their findings revealed a seasonal impact on HEV, with two periods having a statistically significantly increased HEV prevalence level over a one-year period. During the months of March–April and September–October, HEV was more frequent [42]. Also, a group of Spanish researchers investigated HEV seasonality in wild boars in their area and corroborated this seasonal pattern [43]. According to that study, HEV was more prevalent in wild boars in late autumn. Our findings also revealed seasonal changes in HEV detection rate, with the detection rate being highest during months when the ambient temperature fluctuated the most. Because the outdoor temperature is lowest in the winter, pigs huddle close together for warmth. Summer, on the other hand, is the season with the highest outside temperature, and therefore, pigs need to cool themselves. The most typical approach for pigs to cool down is to roll in a moist and wet environment (in small-holdings, this is often in their own excrement). Because this virus is known to be transmitted by the fecal–oral route, we speculate that frequent contact between pigs, particularly between different age groups, could result in an aggressive spread of the virus among the pig population. Nonetheless, in order to confirm our assumptions and reach a final conclusion about the possible influence of seasonal changes on the appearance of HEV in pigs in our country, further intensive research is needed that contributes to the collection and processing of as much data on this topic as possible.

This study suggests a potential risk of HEV cross-contamination on slaughter lines. At all three slaughterhouses, swabs were taken from surfaces that come into contact with pig offal; 22% of these samples were HEV positive. HEV was not detected in surface swabs taken on the slaughter line in Šumadija; however, in Srem, 50% of these surfaces harbored HEV. The results obtained demonstrate a possible association between the presence of HEV on slaughterhouse surfaces and in pig livers because where the HIV detection rate in pig liver samples was higher, the presence of virus in environmental swabs (Srem region) was higher. In Srem, where the proportion of HEV-positive pigs was the highest, the proportion of HEV-positive slaughterhouse surfaces and equipment was also the highest. These results support the need for the strict implementation of good hygienic and manufacturing procedures (good hygienic practices (GHP), good manufacturing practices (GMP)) and HACCP (hazard analysis and critical control point) systems throughout the entire pork production process. In addition, during the cleaning and disinfection processes, it is essential to utilize a disinfection medium that has virucidal properties in addition to its bactericidal properties. Together with employee education and training, strict adherence to the recommended practices could have a substantial impact on preventing new contamination by HEV in the pig production chain.

Several sample categories were tested during this investigation, including liver samples from retail outlets. Five percent of the sixty samples screened from this group were positive for the HEV- 3 virus. Several studies from various nations have reported a comparable prevalence of HEV in retail liver samples. According to Bouwknegt et al. [35], 6.5% of investigated liver samples from pigs in Dutch sales facilities were positive for the presence of this virus. Similarly, according to a group of German researchers, 4% of liver samples from retail establishments tested positive for hepatitis C [33]. Pallerla et al. [44] reported a somewhat higher HEV prevalence in Germany, where 10% of the tested samples (liver products and pork meat products) were positive for HEV, but in Switzerland, the prevalence of HEV in sausages containing liver tissue was 18.9%. [45]. In Italy, Di Bartolo et al. [46] studied the presence of HEV in liver-containing sausages obtained from supermarkets. The incidence of HEV was 22.2% in fresh liver-containing sausages and 4.6% in dried sausages. According to Boxman et al. [47], 14.6% of ready-to-eat sausages in the Netherlands were HEV positive. The occurrence of HEV in RTE food is a concern, particularly for vulnerable populations. Further research is needed to discover the specific infectious dose of HEV, with the ultimate goal of obtaining conclusive answers on the risk assessment of HEV infection caused by contaminated food.

Based on the HEV quantification, the HEV load in the positive liver samples was, on average, 4.41 ± 1.69 log_10_ (2.5 × 10^4^ ± 4.9 × 10^2^) HEV genomic copies per gram of tissue. The number of genomic copies per gram detected in this study ranged from 1 × 10^1^ to 1.4 × 10^9^. Analyzing the scientific papers published so far from across Europe, it can be concluded that the HEV loads (the number of genomic copies per gram of liver tissue) are inconsistent. Therefore, according to Feurer et al. [32], in France, the number of genomic copies per gram of liver tissue ranged from 7.8 × 10^3^ to 1.46 × 10^8^, with an average value of 1.3 × 10^5^. A group of researchers led by Boxman [46] in the Netherlands stated that the interval of HEV genomic copy number per gram in liver tissue ranged from 2.1 × 10^2^ to 1.2 × 10^6^, while the average value was 3.4 × 10^5^. According to that research, the number of HEV copies in products that primarily contained liver tissue was 3 × 10^4^ and 7 × 10^4^, depending on the product group. Furthermore, in the Netherlands, among samples of ready-to-eat sausages originating from supermarkets, the average number of genomic copies per 5 g of sample was 5.7 × 10^2^ [47]. Since the liver tissue in sausages generally does not exceed 50%, the lower numbers of HEVs obtained for this type of sample are completely expected. Also, ready-to-eat European-style sausages go through some heat treatment processes, or at least specific drying processes. All these processes have a negative impact on the survival of HEV in the final product. The findings of our study indicate a possible chance that there is a risk of human infection with HEV from pigs through the consumption of products containing raw liver or insufficiently thermally processed products, as the oral infectious dose of HEV for humans is estimated to be over 10^5.5^ genomic copies of HEV RNA according to French Agency for food, environmental, and occupational health and safety [48].

The sequence analyses performed in this study confirmed that all examined isolates belonged to genotype 3. Within this genotype, 43 HEV sequences clustered unambiguously into the HEV-3a subtype, with nucleotide p-distances ranging from 0.055 to 0.112 to the HEV-3a reference strain (AF082843). On the other hand, two strains were classified into subtype HEV-3c, while one strain (SRB-HEV-25SH-2020|OR147141) was unable to be classified into any of the HEV-3 subtypes. The presence of the HEV-3a subtype was expected, as this subtype was detected in our previous study [26] and in other Central and South Eastern European countries, Hungary [25], Croatia [49], and Slovenia [31]. Generally, genotype 3a is currently the dominant HEV genotype in the Balkans. The geographical spread of this subtype 3a in Serbia could be accounted for by changes in the circulation of HEV strains, likely due to trade of HEV-infected animals from neighboring countries or to the spread of HEV by wild boars. Furthermore, this study showed that Serbian HEV strains have high diversity, were not closely related to other reference strains, and were classified into a separate Serbian subcluster. Unfortunately, data on the distribution of HEV types circulating in the human population in Serbia are still very limited, so further examination of the relationships between human and pig strains is not possible. Further research should be focused on the presence of this virus among people, maintaining a database of HEV strains from people with a focus on linking the routes of zoonotic and foodborne HEV transmission. Genotype 3c is widespread in European countries, such as Germany and the Netherlands [44,47]. In Serbia, most imported piglets and gilts are from these countries (European Commission, 2013), and our hypothesis is that these individual findings of the 3c subtype of HEV are the result of virus transmission through imported HEV-positive pigs from these countries or via wild animal.

## 5. Conclusions

The presence of HEV and its transmission through food has been established as an increasing hazard to human health. This study confirmed that HEV is present among Serbian pigs, with an especially high prevalence in piglets. Our findings revealed potentially seasonal changes in HEV detection rates, with the detection rates being highest during summer and winter. In addition, the discovery of HEV in environmental and retail samples supports the idea that continual animal monitoring should be required in order to decrease the risk of foodborne transmission of this virus to humans. Phylogenetic analysis of HEV genotypes and subtypes confirmed that the 3a subtype is the most common genotype in Serbia. This virus is the appropriate pathogen for fully implementing the One Health strategy, requiring coordination, communication, and collaboration across all domains, including the environment, animal health, human health, and other areas of expertise. If the One Health strategy would be successfully put into practice, all the links in this complex chain would be under control, and the best possible health outcomes would be attained.

## Figures and Tables

**Table 1:** Liver and environmental samples collected from three different regions in Serbia during four seasons in 2019.

Season	Region	Slaughter Lines			Liver Samples from Retail
		Liver Samples		Environmental Swabs	
		<3 Months	6–12 Months		
Winter	Srem	50	25	5	15
	Kolubara	50	25	5	
	Šumadija	50	25	5	
Spring	Srem	50	25	5	15
	Kolubara	50	25	5	
	Šumadija	50	25	5	
Summer	Srem	50	25	5	15
	Kolubara	50	25	5	
	Šumadija	50	25	5	
Autumn	Srem	50	25	5	15
	Kolubara	50	25	5	
	Šumadija	50	25	5	
Total		600	300	60	60
		900			

## Data Availability

Data are contained within the article and Supplementary Materials.

## Supplementary Materials

The following supporting information can be downloaded at: https://www.mdpi.com/article/10.3390/ani14040586/s1, Table S1: Sequence information for pig HEV-3 sequences from three Serbian regions.
