# Supplementary material for: Prevalence, Genetic Diversity, and Quantification of the RNA Genome of the Hepatitis E Virus in Slaughtered Pigs in Serbia
Source: Animals (Basel). 2024 Feb 10;14(4):586. doi: 10.3390/ani14040586 (PMC10886375; doi:10.3390/ani14040586)
Supplement: Supplementary file 1 [file animals-14-00586-s001.zip › animals-2806191-supplementary.pdf]

Table S1. Sequence information for pig HEV-3 sequences from three Serbian regions.

Sequence ID-Collection Date	GenBank acc. Number	HEV-3 Genotype	Region
SRB-HEV-10SH-2020	OR147107	3a	Šumadija
SRB-HEV-110S-2020	OR147109	3a	Srem
SRB-HEV-114K-2020	OR147110	3a	Kolubara
SRB-HEV-114S-2020	OR147111	3a	Srem
SRB-HEV-115SH-2020	OR147112	3a	Šumadija
SRB-HEV-11SH-2020	OR147114	3a	Šumadija
SRB-HEV-121SH-2020	OR147115	3a	Šumadija
SRB-HEV-124K-2020	OR147116	3a	Kolubara
SRB-HEV-128S-2020	OR147117	3a	Srem
SRB-HEV-12K-2020	OR147118	3a	Kolubara
SRB-HEV-131K-2020	OR147119	3a	Kolubara
SRB-HEV-135SH-2020	OR147120	3a	Šumadija
SRB-HEV-143SH-2020	OR147121	3a	Šumadija
SRB-HEV-149S-2020	OR147122	3a	Srem
SRB-HEV-150S-2020	OR147123	3a	Srem
SRB-HEV-153SH-2020	OR147124	3a	Šumadija
SRB-HEV-15R-2020	OR147126	3a	Šumadija
SRB-HEV-160SH-2020	OR147127	3a	Šumadija
SRB-HEV-162S-2020	OR147128	3a	Srem
SRB-HEV-169K-2020	OR147130	3a	Kolubara
SRB-HEV-173K-2020	OR147131	3a	Kolubara
SRB-HEV-173S-2020	OR147132	3a	Srem
SRB-HEV-177S-2020	OR147133	3a	Srem
SRB-HEV-17S-2020	OR147134	3a	Srem
SRB-HEV-185S-2020	OR147136	3a	Srem
SRB-HEV-191S-2020	OR147138	3a	Srem
SRB-HEV-24SH-2020	OR147139	3a	Šumadija
SRB-HEV-25S-2020	OR147140	3a	Srem
SRB-HEV-25SH-2020	OR147141	3	Šumadija
SRB-HEV-27S-2020	OR147142	3a	Srem
SRB-HEV-37SH-2020	OR147143	3a	Šumadija
SRB-HEV-40K-2020	OR147144	3a	Kolubara
SRB-HEV-43S-2020	OR147145	3a	Srem
SRB-HEV-45S-2020	OR147146	3a	Šumadija
SRB-HEV-47R-2020	OR147147	3c	Šumadija
SRB-HEV-48K-2020	OR147148	3a	Kolubara
SRB-HEV-51K-2020	OR147149	3a	Kolubara
SRB-HEV-56S-2020	OR147150	3a	Srem
SRB-HEV-67K-2020	OR147151	3a	Kolubara
SRB-HEV-73K-2020	OR147152	3a	Kolubara
SRB-HEV-76K-2020	OR147153	3a	Kolubara
SRB-HEV-82K-2020	OR147154	3a	Kolubara
SRB-HEV-86S-2020	OR147155	3a	Srem
SRB-HEV-90S-2020	OR147156	3a	Srem
SRB-HEV-95K-2020	OR147157	3a	Kolubara
